# Embedding a user-centred approach in the development of complex behaviour change intervention to improve outcomes for young adults living with type 1 diabetes: The D1 Now Study

**DOI:** 10.12688/hrbopenres.12803.2

**Published:** 2018-08-02

**Authors:** Deirdre M.J. Walsh, Lisa Hynes, Mary Clare O'Hara, Jenny Mc Sharry, D1 Now Young Adult Panel, Séan F. Dinneen, Molly Byrne

**Affiliations:** 1 Health Behaviour Change Research Group, School of Psychology, National University of Ireland, Galway, Galway, H91 EV56, Ireland; 2 School of Medicine, National University of Ireland, Galway, Galway, H91 V4AY, Ireland; 3 SPLAT (Pediatric Lab for Adherence and Transition), West Virginia University, Morgantown, WV, 26506, USA; 4 Research and Development, Strategic Planning and Transformation, Health Service Executive, Dublin 8, D08 W2A8, Ireland; 5 Endocrinology and Diabetes Centre, Galway University Hospitals, Galway, H91 YR71, Ireland

**Keywords:** Behaviour change, Intervention development, Complex intervention, Implementation, Type 1 Diabetes, Young adults, Public and patient involvement

## Abstract

**Background:** Type 1 diabetes (T1D) is an auto-immune condition which requires intensive self-management. Diabetes self-management is challenging, especially during young adulthood. Effective interventions to improve outcomes for young adults (18-30 year olds) with T1D are needed. This paper describes the development of the D1 Now intervention, employing a user-centred approach to engage with stakeholders in parallel with the application of theory.

**Methods: **Intervention development consisted of 4 phases: 1) the formation of a public and patient involvement (PPI) Young Adult Panel (YAP); 2) a systematic review to synthesise evidence regarding the effectiveness of interventions aimed at improving outcomes for young adults with T1D; 3) understand young adults’ diabetes self-management behaviour through engagement with key stakeholders; and 4) an expert consensus meeting to discuss self-management strategies identified in Phase 1 and 3 that would form the core components of the D1 Now intervention.

**Results: **The YAP resulted in meaningful involvement between young adults, researchers and service providers. The systematic review highlighted a lack of quality intervention studies. Qualitative findings highlighted how young adult self-management is driven by complex interactions between external resources, which influence capability, and motivation. The expert panel in Phase 4 highlighted focus areas to improve outcomes for young adults and implementation strategies. Subsequent to these 4 phases, 3 intervention components have been identified: 1) a key worker to liaise with the young adult; 2) an online portal to facilitate relationship building between staff and young adults; and 3) an agenda setting tool to facilitate joint decision-making.

**Conclusions: **This study described the systematic development of an intervention underpinned by theoretical frameworks and PPI, and has identified components for the D1 Now intervention. The resulting intervention content will now be subject to an intervention optimisation process.

## Introduction

Type 1 diabetes mellitus (T1D) is an auto-immune condition with serious short-term and long-term implications. T1D accounts for 5–10% of all cases of diabetes worldwide and its incidence continues to increase ^1^. Self-management of T1D aimed at maintaining optimal glycaemic control is challenging; people with the condition are required to intensively self-monitor blood glucose levels and administer insulin, as well as regulate their diet and exercise ^2^.

Young adults, aged 18–30 years, have been highlighted as being at high risk of poor diabetes self-management ^3^ and suboptimal glycaemic control ^4^. Young adulthood is likely to be a challenging time for diabetes self-management as individuals may struggle to adjust to a number of developmental milestones, including moving away from the family home for the first time, beginning employment or starting university, in addition to transitioning from paediatric to adult healthcare services ^5, 6^. Traditional diabetes care methods may not be the most appropriate for supporting young adults’ self-management. Previous research has shown that often during this period of transition, young adults are at a high risk of disengaging from services that no longer suit their needs ^5^. Young adults rely on adult diabetes services for diabetes-related and emotional support ^7^, yet a number of barriers to young adults’ engagement with adult services have been reported, including differences in the service they experience after transition, a lack of preparation for these differences, and a lack of tailoring of adult services to their individual needs ^8^.

Effective interventions to improve self-management and outcomes for young adults with T1D are needed. There has been relatively little research attempting to develop and test interventions to improve self-management and outcomes for young adults with T1D. A recent systematic review reported that there have been relatively few trials of interventions aimed at improving self-management and the quality of these studies is often poor ^9^.

Recent best practice guidelines for creating and implementing high quality, impactful interventions, have highlighted the need to include key stakeholders such as patients and members of the pubic ^10^. The last decade has witnessed increased emphasis on public and patient involvement (PPI) in population health and health services research ^10^. PPI occurs “when individuals meaningfully and actively collaborate in the governance, priority setting, and conduct of research, as well as in summarising, distributing, sharing, and applying its resulting knowledge” ^11^. User-centred intervention design and development is an approach that involves end-users in designing interventions, and has been shown to increase the usability of interventions ^12^. It has been shown that intervention and service design as well as study recruitment and attrition rates can benefit from employing PPI methodologies ^13– 16^. Thus, involving and empowering patients in health research is increasingly seen as a priority, with the overall aim of increasing the quality, applicability, implementation and impact of research ^17^. However, there are few published examples of fully ‘involved’, co-design approaches to development of health service interventions that involve end-users at every stage of intervention development ^18^.

In parallel with such recommended user engagement and PPI, it is important that intervention design is grounded in theoretical frameworks and principles ^19, 20^. Previous systematic reviews ^21, 22^ have cited how there is currently a lack of specific behavioural theories defining and explaining diabetes-related behaviours. The D1 Now intervention aims to improve outcomes for young adults living with T1D (see official D1 Now website: www.d1now.ie). In developing the intervention, the D1 Now team used best practice intervention design, specifically the Medical Research Council (MRC) guidelines and Behaviour Change Wheel (BCW) frameworks to address the lack of clear evidence in this area. The BCW is a comprehensive framework for incremental intervention development with an emphasis on describing intervention mechanisms and individual behavioural components ^23^. The MRC guidelines define phases of development for optimal intervention design and evaluation of complex interventions ^24– 26^. The BCW can be used in conjunction with the MRC guidelines for the development of complex interventions. Within the BCW is the Capability, Opportunity, Motivation-Behaviour (or COM-B) model that aims to better understand a target behaviour in context. The D1 Now study used initial steps of both the MRC guidelines and the BCW for characterising and designing behaviour change interventions, to systematically develop the intervention content based on a strong evidence base. Specifically, the first step of the MRC guidance (i.e., the development phase) and the initial steps of intervention development using the BCW (i.e., using the COM-B to facilitate behavioural analysis).

This paper describes how we have developed the D1 Now intervention, which aims to improve outcomes for young adults living with T1D. The research was underpinned by a comprehensive approach to PPI while synthesising theoretical and best practice guidelines for intervention development. The paper describes how the research users - young adults with T1D, service providers and policy makers - were involved in a systematic and meaningful way to increase the likelihood of developing a feasible, implementable, applicable and effective intervention.

## Methods

### Phase 1: Formation of a Young Adult Panel (YAP)

Throughout the development work, a core work stream was the formation of the YAP. Full details of the process of the formation of the YAP are published elsewhere ^27^. The panel consisted of 8 young people aged between 18 and 25 years living with T1D who volunteered as co-researchers with the study team. The structured consultation process used to form the YAP commenced in February 2014 and the first official meeting of the YAP was held in June 2014. In line with INVOLVE guidelines ^28^ training was provided in research methods, processes and terminology, in committee skills and in qualitative methods. YAP members have contributed to all aspects of intervention development including reviewing study materials, abstracts, and manuscripts, and conference presentations.

### Phase 2: Reviewing existing literature

In order to establish and synthesise research in this area, a systematic review to explore the evidence regarding the effectiveness of interventions aimed at improving clinical, behavioural or psychosocial outcomes for young adults with T1D was conducted ^9^. This phase is aligned to the first stage of the MRC, the development phase which seeks to identify the existing evidence base. All interventions aimed at improving clinical, behavioural and psycho-social outcomes for young adults with T1D were included. A narrative synthesis (rather than a meta-analysis) was undertaken due to the large degree of heterogeneity between studies. Full methods and findings have been previously published ^9^. The key objectives were to identify components of interventions and to measure the effectiveness of these interventions on outcomes for young adults. Findings were presented to the YAP for assistance with interpreting results prior to publication.

### Phase 3: Qualitative study to understand young adults’ diabetes self-management behaviour

The next phase of intervention development consisted of a qualitative study with key stakeholders to 1) understand the factors which influence diabetes self-management and 2) explore how services and support could be improved. Ethical approval was obtained separately at each hospital recruitment site (NUI Galway Ref: 13/NOV/15, Galway University Hospitals Ref: C.A.1018, NHS National Research Ethics Service Ref: 14/LO/2254 and St. Vincent’s Healthcare Ref: O’Shea/10 Mar 2014). Interviews and focus group scripts were co-produced with YAP members. Semi-structured interviews were conducted with service providers (including doctors, diabetes specialist nurses and dieticians; *n* = 15; for interview guide see Supplementary File 1) and parents of young adults with T1D ( *n* = 10; for interview guide see Supplementary File 2). Three focus groups were conducted with young adults with T1D (Galway focus group n=5; Belfast focus group n= 9; Dublin focus group n= 4; for interview guide see Supplementary File 3). All potential participants were invited to participate via post, apart from service providers who were contacted via email. Potential participants responded to author MCOH to schedule an interview or participation in a focus group. Thematic analysis was used to analyse the data, which was then further categorised using the framework of the COM-B model to identify the factors that drive type 1 diabetes self-management behaviour among young adults (manuscript in preparation; led by author LH).

As described above the COM-B model aims to better understand a target behaviour in context. Therefore, the COM-B model was used to aid identification of appropriate target behaviours for this complex intervention. For example, Capability is the individual’s ability to perform a behaviour (i.e., both physical and psychological). Opportunity describes the specific context that may facilitate the behaviour, while Motivation describes the processes that fuel and influence the behaviour ^23^.

### Phase 4: Information sharing symposium and expert panel consensus meeting

In June 2016, a 3-day event was organised in Galway, Ireland, entitled: ‘Strength in Numbers: Teaming up to improve the health of young adults with type 1 diabetes’ ^29^. The overall aim of the Strength in Numbers event was to exchange knowledge and experiences among stakeholders to gain input into the D1 Now intervention development process. As part of this event, a range of key international and local stakeholders, including young adults with T1D, health professionals, policy makers and researchers, presented on a range of topics relevant to develop effective interventions to improve outcomes for young adults. These included: Supporting self-management; Digital technology for supporting self-management among young adults; Engaging young adults in research and service design; and Innovations in service delivery for young adults with type 1 diabetes. Following this, 18 experts including young adults with type 1 diabetes, researchers, service providers and policy influencers, took part in an Expert Panel meeting. The breakdown of Expert Panel meeting participants illustrates the diversity of roles and perspectives, and is displayed in Table 1.

**Table 1.** Expert panel participants.

Participant categories	Number of representatives
Young adults with type 1 diabetes	4
Psychologists	3
Diabetes Nurse Specialists	2
Doctors	2
Dietitians	2
Policy influencers	3
Researchers	2
**Total**	**18**

Representative groups were formed and each group was assigned a facilitator from the D1 Now research team. One of the authors (MB), an expert in developing complex behaviour change interventions and gathering stakeholder consensus, facilitated this meeting. The findings of the systematic review ^9^ and qualitative study were summarised, and the take home messages from the previous day’s conference were briefly revisited. As a result of the previous phases of the D1 Now intervention development process, three areas emerged as potentially important targets for improving outcomes among young adults with type 1 diabetes. Prior to the meeting, preparatory materials were sent to the Expert Panel members, including a breakdown of the D1 Now study, and descriptions of these three focus areas. The focus areas provided the basis for the Expert Panel meeting activities. The three areas that emerged as potentially important targets for outcomes from Phase 1 and 3 were outlined to experts (see Results section).

Each team was asked to examine and debate two of the three focus areas over two rounds of discussion within each team and with the larger group. Teams were asked to focus on only two of the three focus areas for pragmatic reasons to facilitate in-depth group discussion of the core areas within the allotted short timeframe. Using a structured behavioural analysis recommended as part of intervention development ^23^ each group identified specific strategies which could be used to address their assigned focus area. This involved considering the framework of the COM-B model to identify the factors that drive type 1 diabetes self-management behaviour among young adults and how the suggested strategies would impact on young adults’ ‘Capability, Opportunity and Motivation’ to perform this target behaviour. The strategies identified by each group to address each of the three focus areas were summarised ( Supplementary File 4), which were assessed using ratings of high, medium or low, according to the criteria of; impact on young adult self-management; how possible or feasible the strategy was; and the potential for each strategy to have subsequent positive effects. Each group was asked to generate 3 strategies per session, but time constraints meant this was not achieved in every case.

**Figure 1.**
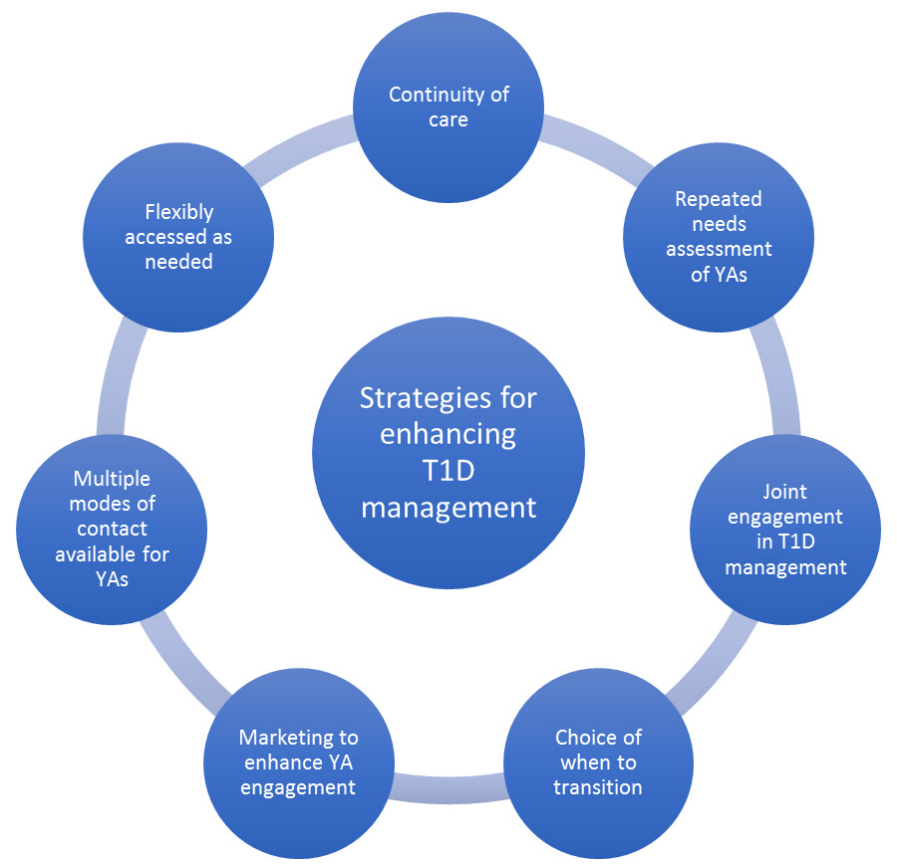
Themes emerging from expert panel discussions.

Expert Panel teams were also tasked with brainstorming plans and barriers for implementing their most promising strategy before presenting to the larger group. These were then grouped to identify relevant themes. Figure 1 in the Results section depicts important themes that emerged during discussion on the plans and barriers to implementation of the promising strategies.

## Results

### Phase 1: Formation of a Young Adult Panel (YAP)

The development of the YAP resulted in meaningful involvement between young adults, researchers and service providers. The YAP made significant contributions to all aspects of the study development, in particular developing the qualitative interview topic guides, the participant invitation letters, consent forms and information sheets, and disseminating study findings’ by submitting scientific abstracts to national conferences, being invited speakers at two national conferences, being interviewed for local newspapers and radio, and by entering national science competitions. The YAP members were part of the organisation committee for our international symposium in June 2016 (see Phase 4) and two YAP members were elected to sit on the study’s steering committee. The D1 Now YAP were named as official Collaborators on a successful Health Research Board (HRB) Definitive Intervention and Feasibility Award to further progress the D1 Now study (Ref: DIFA_2017-034).

### Phase 2: Reviewing existing literature

Eighteen studies were included in the review of interventions aimed at improving clinical, behavioural or psychosocial outcomes for young adults with Type 1 diabetes and categorised as follows: Health Services Delivery (n = 4), Group Education and Peer Support (n = 6), Digital Platforms (n = 4) and Diabetes Devices (n = 4). Study designs included 1 randomised controlled trial, 3 retrospective, 7 feasibility/ acceptability studies and 8 studies with a pre/ post design. Continuity, support, education and tailoring of interventions to young adults were the most common themes across studies. In particular continuity and support have emerged as crucial through other key phases of the current intervention development process. Glycaemic control was the most frequently measured outcome but only 5 of 12 studies that measured it showed a significant improvement. Due to the wide variance of quality and reporting among the studies, the effectiveness of individual interventions outcomes could not be established and warrants further investigation.

### Phase 3: Qualitative study to understand young adults’ diabetes self-management behaviour.

The qualitative engagement study with key stakeholders elicited rich information regarding how self-management of T1D is influenced by interactions between capability, opportunity and motivation. The findings of this study demonstrate that social and physical opportunity drive self-management through access to supportive diabetes service providers which facilitates young adults accessing the health care they want, in a way that is most appropriate and useful to them (i.e., age-appropriate consultation style and frequent follow-up through different modes of contact as well as traditional clinics). Findings suggest that diabetes teams can also optimise external physical and social factors such as access to resources such as diabetes devices and peer networks to enhance self-management. Importantly, an individual’s perception of their capability to self-manage impacts their motivation to perform self-management behaviour in what appears to be a bi-directional relationship. Further, the role of the clinic and the diabetes team were foundational to self-management. According to these findings, all components of the COM-B model play a role in determining self-management behaviour. Physical and social opportunity factors, such as the influence of service providers, emerged as dominant drivers of self-management.

### Phase 4: Information sharing symposium and expert panel consensus meeting

As described in the Methods section, during the Expert Panel meeting, each team was asked to examine and debate two of the three focus areas below over two rounds of discussion within each team and with the larger group. Using a structured behavioural analysis recommended as part of intervention development ^23^ each group identified specific strategies which could be used to address their assigned focus area.

These focus areas to improve self-management were:

***1. The way young adults are introduced to the adult diabetes clinic***
*The experiences young adults have around the time they begin to attend adult diabetes clinics play a major role in determining how well they adjust to having and managing their diabetes during young adulthood.*
***2. Attendance at diabetes clinic appointments and contact between appointments***
*Addressing the system for making appointments and improving ways for young adults to communicate with service providers outside appointments are important targets for making it easier for young adults to engage with the diabetes clinic, and to access the diabetes education and other support available in the clinic.*
***3. Building relationships between young adults and service providers***
*According to previous research* ^5^ *having a relationship with at least one service provider in adult diabetes clinics is important for self-management as it helps to ensure that young adults’ experience a service which is tailored to their needs.*

The full list of strategies identified by each group to address each of the three focus areas can be seen in Supplementary File 4. The strategies deemed to be most promising for addressing each focus area (printed in bold) according to the Expert Panel are listed in Table 2.

**Table 2.** Expert panel identified strategies and proposed operationalisation.

Strategy	Proposed operationalisation
**1. The way young adults are** **introduced to the adult** **diabetes clinic**	• Recruit a ‘named supporter’ to the young adult diabetes team, who may not be an expert in type 1 diabetes to advise and guide young adults through the process of settling into the adult diabetes clinic.
	• Launch a website and system of email contact to introduce young adults to the clinic, including logistical information and information about members of staff, and to facilitate contact between young adults and the clinic staff as needed.
**2. Attendance at diabetes** **clinic appointments** **and contact between** **appointments**	• Create an online appointment booking system and pre-consultation agenda-setting tool to support engagement among young adults with clinic appointments and contacts between appointments, and proactive follow-up by service providers following missed appointments.
	• Diabetes service providers will communicate with young adults to discuss and agree upon the purpose of clinic appointments to ensure the clinic is up-to-date with the needs and circumstances of young adults and to clarify the expectations of young adults and service providers. This will be carried out during an initial consultation and reviewed online and face-to-face, as required.
**3. Building relationships** **between young adults** **and service providers**	• Create an agenda-setting tool to be used before and during consultations to facilitate relationship development and collaborative diabetes management between young adults and service providers. The pre-consultation activity could be carried out online.
	• Recruit a youth worker and integrate them within the diabetes team to bridge the gap between the young adult and service providers. This person may not have a background in diabetes management but will be an expert in working with young people, who will proactively reach out to young adults and will take a holistic approach to addressing the need of young adults.

Results of Expert Panel team discussions on plans and barriers for implementing promising strategies are depicted in Figure 1. Seven key areas were identified: 1) strategies to facilitate continuity of care, which was seen as important for relationship building within the therapeutic relationship and highlights the desire to work with a healthcare professional who is familiar with each individual and their history; 2) the needs of young adults should be frequently reviewed, emphasising the nature of young adulthood as a time of transition which requires the healthcare team takes a dynamic approach to adapt to changing demands; 3) joint engagement of both young adults and service providers in diabetes management. Joint engagement in diabetes management requires collaborative discussions whereby both young adults and healthcare professionals outline current care needs and future goals and action plans on a regular basis; 4) a choice of when to transition from paediatric services and to which clinic in the adult service. Young adult involvement in the transition process ensures that there is limited stress experienced and enables the young adult to adapt to the expectations associated with attending an adult service in a way that is appropriate for them; 5) use strategies from relevant arenas (i.e., social psychology and marketing) to engage young adults. Services should leverage more contemporary means of engaging with this younger cohort. Traditional methods of initiating engagement (e.g., appointment letters) may not be the most appropriate for this target group; 6) multiple modes of contact and engagement should be available. A variety of modes of communication are now needed to ensure that the care needed is delivered as efficiently as possible. This could potentially include text messages and other online services to complement more traditional modes of communication; and 7) clinics designed to be accessed by young adults flexibly, as and when they are needed. This key area of accessibility is very important for this cohort. Flexibility is integral to this group due to the constant changing needs (e.g., moving out of home for the first time, work/study schedules, travelling abroad alone) and healthcare professionals need to have the capacity and methods to respond and deliver care as needed.

There was significant overlap in the details of the implementation plans described across the groups. According to the Expert Panel, the most promising strategies for addressing the focus areas will involve action on the part of the diabetes service organisation, service providers and young adults, often in partnership. The philosophy and running of the clinic will need to change, for example staff must be open to integrating new approaches such as online systems, communicate in new ways and adopt a young adult-centred ethos. Flexibility is the theme which describes panel members’ consensus around when, where and how often a strategy should be implemented. According to the Expert Panel, strategies such as meeting with a key worker or youth worker should occur at the beginning of an intervention and intermittently as required thereafter in a personalised manner and when the young adult requires the support. Platforms or locations for connecting with key workers, such as online or in person, in the clinic or another suitable location, should also be utilised. Bringing everyone to the table to implement strategies effectively was also a strong theme across groups and focus areas. Successfully implementing strategies will involve the participation of all stakeholders including young adults and service providers and depending on the strategy and the preferences of young adults, it may also involve friends and family.

The barriers to implementing each strategy included funding, acceptance and engagement, training of service providers and young adults, logistical issues such as planning, time, staffing levels and access to technology within the HSE, identification or recruitment of appropriate key workers and accountability of key workers. Solutions for overcoming barriers included engaging with Diabetes Ireland (Irish national charity) and with young adults themselves, for example learning from other service-user groups seeking similar changes in other services, referring to existing best practice guidelines, and pilot testing strategies and collecting feedback. In addition, it may be beneficial to seek funding from other sources to support this work or aspects of it, such as Social Entrepreneurs Ireland [national group aimed at solving big issue societal problems). Issues related to engagement among young adults and service providers could be addressed using marketing and social psychology approaches, by building flexibility within strategies and addressing technology gaps (e.g., leveraging existing IT solutions and services to deliver care in a more nuanced way).

### D1 Now: Current proposed intervention components

Subsequent to the above development process, three components of the D1 Now intervention have been identified: 1) a key worker to introduce the young adult to the diabetes service and flexibly address the needs of young adults within the clinic; 2) an online Young Adult Service Portal to facilitate relationship building between staff and young adults; and 3) an agenda setting tool to facilitate joint decision making and goal-setting within clinic appointments. These three components are currently being refined within the intervention optimisation phase. The operationalisation of these components for the final intervention content will be developed based on iterative cycles of pilot testing and feedback from young adults and healthcare professionals. The final version of the D1 Now intervention will then be subject to feasibility and piloting phases (funded by a HRB Definitive Intervention Feasibility Award).

## Discussion

We have mapped the incremental phases adopted by the D1 Now research team to systematically develop an intervention to improve outcomes for young adults with type 1 diabetes. This paper addresses a clear gap in highlighting the key steps of intervention development, in particular where PPI is integrated at each step and key stakeholder recommendations are incorporated in a tangible way within the intervention. We have outlined how we engaged meaningfully with all stakeholders including young adults with T1D, service providers and policy makers for T1D, in order to increase the likelihood of developing a feasible, implementable and effective intervention.

The PPI approach was instrumental in moving from theory-based concepts to operationalising core intervention components in concrete terms. The use of this phased approach to identify acceptable and feasible ways of operationalising theoretical constructs from the BCW is crucial. In particular, the COM-B and behavioural analysis aspects informed the initial development phase of the D1 Now intervention components. This ensures that the intervention content and method of delivery is context-specific and appropriate for the provision of care to young adults with T1D. This paper extends behavioural science methodology by utilising best practice (i.e., MRC and BCW frameworks in new contexts).

### Reflections on the user-centred approach within D1 Now intervention development

Engaging with key stakeholders is recommended as best practice for effective intervention development ^10, 13^ and researchers are increasingly encouraged to adopt a PPI framework within health research. The D1 Now study team has demonstrated that it is both feasible and desirable to include a PPI panel of young adults with T1D in health research ^27, 29^.

Formation of the YAP was time-intensive and required commitment from the research team to drive recruitment and identified training with the YAP members. Acting as a member of the YAP required regular meetings, up-skilling as many members became familiar with new terms and reading different types of material (participant information sheets, grant applications and manuscripts being prepared for submission) and engagement with an iterative research process. This involved managing expectations of both the YAP and the research team in terms of what is feasible, and implementable, and how quickly change can happen given research designs and processes. Expectation management was undertaken by a neutral ‘knowledge broker’ Jigsaw [a youth mental health service with experience in young adult panels] ^30– 32^. The process of understanding and integrating the ethos of PPI occurred over the course of the study. The trust between the research team and the YAP grew through multiple interactions, culminating in the large scale event described in Phase 4 (Information sharing symposium and expert panel consensus meeting). Currently, a sub-study aiming to explore the impact of PPI on the intervention development and overall study is underway.

### Implications for research and practice

It has become clear that D1 Now needs to reimagine T1D young adult care from its very foundation with the guidance of the YAP ^9, 33^. Therefore, an exploratory approach underpinned by best practice and theory was chosen to address the aims of this study. This user-centred approach acts as a template for other research teams to work collaboratively with people living with a chronic condition to develop meaningful strategies for impacting health services.

The current study highlights the importance of consistent engagement with key stakeholders throughout intervention development. Theory (e.g., the BCW) is an important tool used throughout this process but all theory and interventions require grounding within a health services context if they are to contribute to successful service re-design. Our PPI approach is particularly important to identify potential barriers to implementation within the T1D population.

Use of the BCW, and identifying aspects of capability, opportunity and motivation that should be targeted will facilitate explicit linking of these ‘drivers’ of behaviour to intervention functions which will allow us to explicitly outline the proposed mechanisms of action of the D1 Now study intervention. Investigating these hypothesised mechanisms of action, and whether included BCTs are appropriate and acceptable for our target population, will be further examined within future testing and refining phases and subsequently with a feasibility phase and randomised pilot.

### Strengths and limitations

The systematic intervention development process addressed an important criticism of previous behaviour change interventions targeting complex self-management behaviours. As identified by O’Hara and colleagues ^9^, previous interventions are poorly described and their effectiveness cannot be accurately evaluated. However, a phased intervention development process is hugely resource-intensive ^34^. Various outputs, individual and team decisions and decision logic can be difficult to document and present in a coherent reusable fashion. The use of the BCW framework along with checklists such as the Template for Intervention Description and Replication (TIDieR) checklist ^35^ to report the intervention content will assist in future replication and prevent recurring research waste as demonstrated through previous reviews of interventions unable to yield tangible effective intervention mechanisms to be implemented in future research.

A further strength of this study, was the multidisciplinary nature of the stakeholders and the PPI approach adopted throughout the D1 Now study. This facilitated discussion between allied health professionals, patients and researchers which resulted in rich and nuanced output to explore within the next phase of the D1 Now feasibility and pilot phases.

## Conclusions

We have described the systematic development of a multifaceted behaviour change intervention with reference to theoretical frameworks, and PPI, and have identified core intervention components. All phases contributed meaningfully to the intervention development process by using a user-centred approach that focuses on understanding and accommodating the perspectives of the people who will ultimately use the intervention. This is one of the few published examples of a fully co-design approach within an Irish health-service intervention. The resulting intervention content will now be subject to an iterative and co-development process to test and refine the intervention that is grounded in a systematic, rigorous, in-depth understanding of the psychosocial context of young adults with T1D. It will then be formally tested in a feasibility and a randomised pilot before evaluating the fully operationalised D1 Now intervention in a definitive trial.

## Consent

Where applicable within the current manuscript, written informed consent for publication of the participants’ data was obtained.

## Data availability

Qualitative transcripts from Phase 3 are unavailable for sharing in full as the current study REC approval does not include permission for sharing of qualitative data. A manuscript detailing an in-depth account of Phase 3 is currently in preparation and selected quotes will be contained within this manuscript (this is led by author LH).

## Supplementary material

Supplementary File 1: Interview guide for Diabetes Healthcare professionals.

Supplementary File 2: Interview guide for Friends/ Family of Young Adults with Type 1 Diabetes.

Supplementary File 3: Interview guide for Focus Groups with Young Adults (18–25 years) with Type 1 Diabetes.

Supplementary File 4: Strategies identified by expert groups during consensus meeting.
