# Supplementary material for: Embedding a user-centred approach in the development of complex behaviour change intervention to improve outcomes for young adults living with type 1 diabetes: The D1 Now Study
Source: HRB Open Res. 2018 Aug 2;1:8. Originally published 2018 Feb 28. [Version 2] doi: 10.12688/hrbopenres.12803.2 (PMC6973524; doi:10.12688/hrbopenres.12803.2)
Supplement: Supplementary file 1 [file hrbopenres-1-13926-s0000.tgz › f39902a2-4f30-4497-ad82-cf31b84d6e18.docx]

**Qualitative Topic Guide**

**Interview with Diabetes Healthcare Professionals**

*Aim: to describe an optimum lifestyle for a young adult with T1D*

*Someone’s health and their health outcomes does not depend on whether they attend a service or not. It is far more complex. In the first instance, we want HCPs in the interviews to:*

1. **Think About You and Your Relationship with Young Adults with T1D**

- As a HCP what do you think your role in diabetes care should ideally be?
- Do you feel that you are always able to give this level of care? If not, what prevents this level of care being delivered?

If not, please describe the barriers

- those delivering care?
- patients themselves?
- health care system?
- How do you think you’d cope if you were diagnosed with type 1 diabetes?
- If you got a blank canvas, can you please describe the optimum lifestyle for a young adult with T1D
- If you got a blank canvas, can you please describe an optimum care pathway for a young adult with T1D
- Newly diagnosed patients
- Patients new to this service
- How would you describe your relationship with young adults in clinics?
- Have your relationships with young adults with T1D have changed over the year. If so, in what way and why?
- Describe if you feel your relationship with young adults differs to your relationship with other patient groups you see with type 1 diabetes?
- What in your opinion seems to be the most important issues for young adults you see in clinic?

1. **Diabetes Care**

- In your opinion, what are the most important changes in the diabetes healthcare landscape in the recent years?
- Are there still changes you’d like to see take place?
- What aspects of the current diabetes service are you most confident/ happy with?
- What aspects of the current diabetes service frustrate you most?

1. **Diabetes Self Management Support**

**Education**

- Describe any courses/ programmes/ workshops you have completed that you feel have improved your care for patients with T1D

**Family/ Friends**

- In your opinion describe the role of family members and friends in supporting someone who have T1D?
- Is there a role for GP/ primary care in supporting someone with T1D?

**Technology**

- Technological advances try to make life easier for patients living with diabetes but from the HCP point of view, what advances aid your practice best?
- Are there app/ websites that you would recommend to patients with T1D?
- Are there app/ website that you would recommend to other diabetes HCP?
- Are there any novel devices/ apps/ online services that you are aware of that you think could benefit patients with T1D that are not currently available in Galway/ Ireland?

1. **Improving Diabetes Care**

- How would you improve type 1 diabetes care in this centre?
- Finally, we’ve covered topics on your relationship with young adults with T1D, on diabetes care, on self management, support and technology but is there anything you would like to add, suggest or comment on?
