# Supplementary material for: Embedding a user-centred approach in the development of complex behaviour change intervention to improve outcomes for young adults living with type 1 diabetes: The D1 Now Study
Source: HRB Open Res. 2018 Aug 2;1:8. Originally published 2018 Feb 28. [Version 2] doi: 10.12688/hrbopenres.12803.2 (PMC6973524; doi:10.12688/hrbopenres.12803.2)
Supplement: Supplementary file 2 [file hrbopenres-1-13926-s0001.tgz › 141971c8-d254-4935-8e0b-732b307bd640.docx]

**Qualitative Topic Guide**

**Interview with Friends/ Family of Young Adults with Type 1 Diabetes**

*Aim: to describe an optimum lifestyle for a young adult with T1D*

*Someone’s health and their health outcomes does not depend on whether they attend a service or not. It is far more complex. In the first instance, we want friends/ family of young adults with type 1 diabetes in the interviews to:*

1. **Introduction**

- Name
- Name of young adult with type 1 diabetes
- How long have you known them?
- How long have you known they have diabetes?

1. **Background**

- Prior to XXX telling you that she/ he had diabetes, describe what you knew about diabetes?

or

- Describe what you knew about diabetes before XXX told you she/ he had diabetes?
- Describe if you knew any differences between type 1 and type 2 diabetes?
- Describe if you had ever seen anyone test blood glucose levels or inject insulin prior to XXX being diagnosed?

1. **Diagnosis of Your Friend/ Family Member with T1D**

- What was your reaction when you XXX was diagnosed?
- Describe how the diagnosis impacted you/ your life, if at all?
- Describe how you think you would manage if you had diabetes?
- Since XXX was diagnosed, please describe how your own understanding of the condition has changed?
- How did you feel and cope with the diagnosis?

1. **Think About You and Your Relationship with Young Adults with T1D**

- Describe if you feel that XXX having diabetes has impacted your relationship?
- Describe how having someone with type 1 diabetes in the family influence the day-to-day activities / eating habits/ lifestyle of the family?
- In your opinion describe if some family members made allowances or adaptations for XXX and if the whole household did likewise? Were there differences between family members in the treatment of XXX?

or

- Describe if having a friend with type 1 diabetes influences the day-to-day activities of the group, e.g., organising social events, sporting events, holidays etc…?
- Knowing that XXX was coming along to an event, e.g., cinema, a meal, a holiday, night out, did you ever have to make allowances/ adaptations to your plans?
- By knowing someone with T1D has your view on the condition been affected?
- Describe if there has ever been a time (or can you think of a scenario) where having T1D has limited you from doing something?
- (If a sibling): How did you feel when XXX was diagnosed? Were there adaptations the whole household had to make? Did you ever feel your issues were not as important or given the same time as XXX? Did you ever feel sidelined?

1. **Supporting the Person with Type 1 Diabetes**

- Describe if you were ever worried about XXX when they were leaving home for the first time, e.g., moving away to college, going travelling, going away to work, when they started to drive, when they started to drink?
- What are your main concerns for XXX having diabetes?
- Describe if you have ever had to intervene medically, e.g., insists they check their blood glucose, eat something, call a ambulance, call a parent/ guardian, inject glucagon?
- If so, how did that make you feel?
- Would you realise if XXX had a high/ low blood glucose level? If so, describe the symptoms?
- If you have observed XXX symptoms of high/ low blood glucose levels, how did it make you feel?
- Describe if you ever had to advocate on behalf of XXX where their diabetes care is concerned, e.g., with a GP, consultant, nurse etc…?
- Do you feel involved in the self management/ support of XXX with their diabetes? If so, please describe the role

1. **Technology**

- Describe if you have noticed any changes to blood glucose meters, needles and insulins over the years?
- Please describe any online supports/ apps for people with type 1 diabetes that you are aware of?
- Are you aware of any apps/ online supports for friends/ family member of those with T1D? If so, please describe
- Can you think of any other online supports/ apps that might be useful for people with type 1 diabetes?

1. **Diabetes Clinic**

- Have you ever attended a clinic appointment with XXX?
- If so, please describe this experience?
- Describe if XXX has ever complained about the care they receive from their diabetes team?
- Describe if XXX has ever praised the care they receive from their diabetes team?
- If you have attended a Diabetes Clinic, what were experiences? Have you any thoughts on how you might change/ improve it?
- Looking back to when XXX was diagnosed/ XXX told you about her/ his diagnosis, what supports/ advice could you have benefited from?
