# Supplementary material for: Embedding a user-centred approach in the development of complex behaviour change intervention to improve outcomes for young adults living with type 1 diabetes: The D1 Now Study
Source: HRB Open Res. 2018 Aug 2;1:8. Originally published 2018 Feb 28. [Version 2] doi: 10.12688/hrbopenres.12803.2 (PMC6973524; doi:10.12688/hrbopenres.12803.2)
Supplement: Supplementary file 3 [file hrbopenres-1-13926-s0002.tgz › e40ac643-11b3-422f-84d5-b53fd505ad00.docx]

**Qualitative Topic Guide**

**Focus Groups with Young Adults (18-25 years) with Type 1 Diabetes**

*Research proposal*

*The purpose of the qualitative research is to:*

1. *understand the focus, process and context of care delivery for young adults with type 1 diabetes*
2. *understand what young adults with type 1 diabetes mean by an optimal lifestyle for supporting good control and self-management*
3. *explore ways of improving diabetes services for young adults to inform the composition of the intervention by seeking their advice on what is feasible in practice.*

*Someone’s health and their health outcomes does not depend on whether they attend a service or not. It is far more complex. In the first instance, we want young adults in the focus group to:*

1. **Introduction**

- Name?
- Where do you live?
- How long have you had T1D?

1. **Think About Themselves**

- In general, describe the optimal health for a young person? Describe an optimum lifestyle for a young adult with T1D

1. **Understanding Diabetes**

- If you’re comfortable talking about it, describe if you think it is possible to get used to having diabetes? E.g., Does it take long to accept/ adjust?
- How would you describe your current understanding of diabetes?
- How do you feel about the level of understanding you have about diabetes?

1. **Your Diabetes Care and how you manage it**

*People with T1D have to manage their diabetes everyday. How people do this is called ‘self management’, it refers to the fact that they have to test their blood glucose levels frequently through the day (before meals, pre- and post-exercise, if they are feeling low etc…), they have to estimate the amount of carbohydrate they eat and calculation the appropriate amount of insulin to take for that meal and know how to take corrective amount of insulin.*

1. What are the things you need to do to manage your diabetes on a day-to-day basis?
2. What aspects of your self care do you struggle with/ find difficult?
3. What aspects of your self care do you find straightforward?
4. What could be done to improve your self management (by yourself/others)
5. Thinking about your current insulin regime and insulin devices – describe if you are happy with it or how it could be improved?
6. What health outcome do you want to achieve, or is most important to you right now?
7. What would help you manage it better?
8. **Self Management Support from Education**

- Have you attended any education programme aimed at diabetes self management?
- What were helpful/ least helpful aspects of this programme?

**Self Management Support from Other People**

- Describe the various supports available to you to help you manage your diabetes? E.g., family, friends, colleagues, HCP, websites, apps
- Have you ever spoken to anyone e.g., GP, clinic staff or psychologist about what it’s like to have diabetes, and the way you feel about having diabetes?
- What methods of communication do you use most frequently with family/ friends?
- What methods of communication do you use most frequently with HCPs?

**Self Management Support from Technology**

- Describe what apps/ website in general you visit most frequently?
- Are any of them related to diabetes/ diabetes support?
- Describe any technology that you currently use in managing their diabetes? E.g., glucose monitors, online supports, apps?
- Can you think/ describe any online/ app supports that could better help you manage your diabetes?
- Can you think/ describe any online/ app supports that could better help you engage with your diabetes team/ clinic/ diabetes services?

1. **Current Relationships with Healthcare Professionals**

- How would you describe your current relationship with your diabetes care team?
- How would describe the care you currently receive in the hospital for your diabetes?
- How do HCPs treat you (as a person or just your diabetes)?
- Do your feel your health care team listen to you?
- What aspects of HCP care and support do you value most?
- How do you think HCP care could be improved?
- Do you think HCPs understand the impact of diabetes on your lifestyle?
- What is the main focus of the conversations you have with HCPs during your consultations in the diabetes clinic? E.g., are there other things you feel are more important to your health or management that you’d like to discuss or are not?
- Describe a typical visit to the Diabetes Centre, in terms of who you see? Do you see the same people? Do you feel you always need to see all the HCPs you see at clinic?

1. **Improving Care**

- How would you improve your diabetes clinical care?
- What would you change?
- What other supports were helpful to you at the time of your diagnosis? What would have been helpful around the time of diagnosis?
- Is there anything I’m forgetting, do you have any other ideas for improving your diabetes care and your engagement with your diabetes care team?
