# Supplementary material for: Embedding a user-centred approach in the development of complex behaviour change intervention to improve outcomes for young adults living with type 1 diabetes: The D1 Now Study
Source: HRB Open Res. 2018 Aug 2;1:8. Originally published 2018 Feb 28. [Version 2] doi: 10.12688/hrbopenres.12803.2 (PMC6973524; doi:10.12688/hrbopenres.12803.2)
Supplement: Supplementary file 4 [file hrbopenres-1-13926-s0003.tgz › 19c842b1-dfdb-435d-ad9c-6f82689a7c3c.docx]

**Supplementary File 1: Strategies identified by expert groups during consensus meeting**

Expert Panel Group Work: Session 1

| *Group 1 Focus Area: The way young adults are introduced to the adult diabetes clinic* | | | |
| --- | --- | --- | --- |
| Possible strategies | Impact of strategy on young adult self-management | Likelihood that the strategy is possible | Potential positive knock on effects |
| 1. Information pamphlet to introduce young adults to the adult service | Low | High | Medium |
| **2. Named supporter/ key worker to support the young adult from transition** | **Medium/High** | **High** | **High** |
| *Group 2 Focus Area: Attendance at diabetes clinic appointments and contact between appointments* | | | |
| 1. Flexible online booking system | Low/Medium | High | High |
| 2. Motivational consultation to discuss expectations & reach agreement | High | Low | High |
| **3. Appointment reminders & proactive agenda-setting** | **Medium** | **High** | **High** |
| *Group 3 Focus Area: Building relationships between young adults and service providers* | | | |
| **1. Youth worker** | **High** | **Medium** | **High** |
| 2. Asking one question | High | High | Low/Medium |
| 3. Choosing staff member for appointments | High | Medium | High |

Expert Panel Group Work: Session 2

| *Group 1 Focus Area: The way young adults are introduced to the adult diabetes clinic* | | | |
| --- | --- | --- | --- |
| Possible strategies | Impact of strategy on young adult self-management | Likelihood that the strategy is possible | Potential positive knock on effects |
| **1. Online clinic introduction & communication system** | **High** | **High** | **High** |
| *Group 2 Focus Area: Attendance at diabetes clinic appointments and contact between appointments* | | | |
| 1. Phone call prior to clinic | High | Medium | Medium |
| **2. Agreeing on the purpose of clinic appointments with young adults** | **High** | **Medium** | **High** |
| 3. Prepare for consultation | High | High | High |
| *Group 3 Focus Area: Building relationships between young adults and service providers* | | | |
| 1. Young adult health records | High | High | Medium |
| 2. Find out about team online before clinic appointment | Low | High | Medium/High |
| **3. Agenda-setting package/tool** | **High** | **High** | **Medium/High** |
