# Supplementary material for: Embedding a user-centred approach in the development of complex behaviour change intervention to improve outcomes for young adults living with type 1 diabetes: The D1 Now Study
Source: HRB Open Res. 2018 Aug 2;1:8. Originally published 2018 Feb 28. [Version 2] doi: 10.12688/hrbopenres.12803.2 (PMC6973524; doi:10.12688/hrbopenres.12803.2)
Supplement: Peer review history

# Peer review: Reviewer response for version 1

**DOI:** 10.21956/hrbopenres.13863.r26226

**Published:** 2018-06-26

**Authors:** Jackie Sturt, Mette Due-Christensen

**Affiliations:** 1 Florence Nightingale Faculty of Nursing, Midwifery & Palliative Care, King's College London, London, UK

Thank you for inviting us to review this paper. This paper aims to develop an intervention for young adults living with type 1 diabetes using both user-centred design methods and behavioural and psychological theory. The intervention hopes to support their self-management and increase their engagement with diabetes services.

 We think this is a really well written paper and outlines the process very well. We agree with the points the 1 ^st^ reviewer has made especially regarding more description of the themes in the results section.

 The proposed logic model for their intervention is, we think, more broadly applicable to other long term conditions. It resonates with evidence found in the LYNC study ^1^ relating to how young adults with many different mental and physical health long-term conditions including type 1 diabetes use, and perceive benefit from, digital consulting and the therapeutic relationships that it seems to facilitate.

 It would be helpful to the reader if young adulthood was defined in terms of age span in the abstract.

 It is also important to note that type 1 diabetes is not most commonly diagnosed in children and adolescents but throughout the adult life span. ^2^^,^^3^

We confirm that we have read this submission and believe that we have an appropriate level of expertise to confirm that it is of an acceptable scientific standard.

## References

[1] : Timely Digital Patient-Clinician Communication in Specialist Clinical Services for Young People: A Mixed-Methods Study (The LYNC Study). *J Med Internet Res*.2017;19(4) : 10.2196/jmir.7154 e102 10.2196/jmir.7154 28396301PMC5404145

[2] : Frequency and phenotype of type 1 diabetes in the first six decades of life: a cross-sectional, genetically stratified survival analysis from UK Biobank. *The Lancet Diabetes & Endocrinology*.2018;6(2) : 10.1016/S2213-8587(17)30362-5 122-129 10.1016/S2213-8587(17)30362-5 PMC580586129199115

[3] : Incidence of type 1 and type 2 diabetes in adults and children in Kronoberg, Sweden. *Diabetes Res Clin Pract*.2008;82(2) : 10.1016/j.diabres.2008.07.022 247-55 10.1016/j.diabres.2008.07.022 18804305

# Peer review: Reviewer response for version 1

**DOI:** 10.21956/hrbopenres.13863.r26116

**Published:** 2018-03-26

**Authors:** Beth Pyatak

**Affiliations:** 1 Chan Division of Occupational Science and Occupational Therapy, University of Southern California, Los Angeles, CA, USA

This article clearly outlines and explains an iterative process of intervention development addressing healthcare engagement among young adults with type 1 diabetes which was informed by theory, existing literature (systematic review), and key stakeholder input. Furthermore, the intervention development process was informed by meaningful involvement of end users (young adults with type 1 diabetes) through the formation of a Young Adult Panel. The researchers are to be commended for their approach to integrating multiple perspectives in intervention development in a process that merges both best research evidence and practical feedback from stakeholders and users. The study is clearly and accurately described, cites appropriate previous literature, and clearly outlines reasons for source data being unavailable to the public. A few minor points of clarification would help to strengthen the manuscript even further, as outlined below:

 In the Methods section:

- Can you explain why each team asked to examine only two of the three focus areas? My assumption is that it was for pragmatic reasons, but this or any other rationale should be briefly outlined.
- Can you briefly clarify/explain the structured behavioral analysis approach that was used to identify strategies for each focus area?
- The term "knock on" is not familiar to this (U.S.-based) reviewer. Could another term be used in its place or could this term be briefly defined?

In the Results section:

- The themes which emerged from the expert panel discussions are shown in Figure 1 and reviewed in the text, but only very briefly/concisely. It would help the reader better understand these themes if they could each be described/elaborated in one or two sentences. 

I confirm that I have read this submission and believe that I have an appropriate level of expertise to confirm that it is of an acceptable scientific standard.
